# Editorial to “Feasibility and efficacy of 50 W ablation with the TactiFlex catheter for the initial pulmonary vein isolation of atrial fibrillation”

**DOI:** 10.1002/joa3.13215

**Published:** 2025-01-21

**Authors:** Naoto Otsuka, Yasuo Okumura

**Affiliations:** ^1^ Division of Cardiology, Department of Medicine Nihon University School of Medicine Tokyo Japan

**Keywords:** atrial fibrillation, high power short duration ablation, pulmonary vein isolation

Radiofrequency (RF) ablation has become an established primary treatment for atrial fibrillation (AF), with pulmonary vein isolation (PVI) as the cornerstone of therapy. Multiple RF ablation catheter options and strategies are available, including high‐power short‐duration ablation using 50 W output. Recently, an innovative contact force (CF) sensing catheter featuring a mesh‐shaped irrigation tip (TactiFlex™ SE, Abbott) has been introduced into clinical practice. However, clinical data remain limited, and the safety and efficacy of this catheter in real‐world practice have yet to be fully clarified. In this article,^1^ Matsumoto et al. evaluated the clinical safety and efficacy of the TactiFlex catheter for AF treatment. Their study reported that first‐pass PVI was achieved in 82% of right pulmonary veins (RPV) and 87% of left pulmonary veins (LPV) in 100 AF patients, including those with paroxysmal AF (PAF) and non‐PAF. Ablation parameters included a CF range of 5–20 g, an ablation duration of 15–20 s, and a fixed power of 50 W. The authors adjusted ablation duration based on impedance measurements; for pre‐ablation impedance >120 ohms, the duration was 20 s, while for lower values, it was set at 15 s. The study reported only one gastric hypomotility following left atrial box ablation, with no fatal complications, including steam pops. The authors also identified a cut‐off value for impedance drop to achieve first‐pass isolation: 13.5 ohms for LPV and 14.5 ohms for RPV. This finding underscores the importance of monitoring impedance drop to achieve effective PVI. A previous study has shown that approximately 80% of steam pops occur when the impedance drop exceeds 18 ohms.^2^ Interestingly, the threshold impedance drop for achieving first‐pass isolation in this study (13.5–14.5 ohms) is close to the reported cut‐off for preventing steam pops (18 ohms). While the risk of steam pop remains a concern, the TactiFlex catheter appears to have a significantly lower rate of steam pops compared to the SmartTouch Surround Flow (STSF) catheter (1.7% vs. 65.7% at 50 W).^3^ This lower risk is attributed to the catheter's unique mesh‐shaped irrigation tip, which enhances irrigation efficiency and reduces the risk of char and thrombus formation. One year after ablation, the AF‐free rates were 81.7% in the PAF group and 76.3% in the non‐PAF group. Among the 16 patients who experienced AF recurrence, 10 underwent a second ablation session. Of these, two had PV reconnection, five had residual potentials in the carina region without PV reconnection, and three had neither. These results demonstrate the durability of PVI lesions created with the TactiFlex catheter over the long term. Notably, the absence of significant differences in first‐pass isolation rates between attending physicians and fellows (RPV: 81.3% vs. 83.3%, *p* = 0.839; LPV: 93.8% vs. 85.7%, *p* = 0.381) highlights the catheter's user‐friendly design and reliability.

Compared to the STSF catheter, the TactiFlex catheter produced smaller lesions across all parameters (lesion depth, maximum surface diameter, surface area, lesion volume) when operating at 50 W for 20 s.^3^ Despite the smaller lesion size, the TactiFlex catheter achieved effective transmural ablation, as the mean lesion depth of 3.8 mm was sufficient to penetrate the thin walls of the pulmonary veins and left atrium, which measure approximately 0.88–1.83 mm on computed tomography.^4^ The high PVI durability rates observed at 1 year further confirm the adequacy of these lesions.

The TactiFlex catheter combines the ability to create effective lesions for durable PVI with enhanced safety, including a reduced risk of steam pops, esophageal injury, and phrenic nerve damage. These advantages are largely attributed to its unique irrigation system and mesh tip design. However, the incidence of severe adverse complications, such as tamponade and stroke, has significantly decreased in recent years, so the number of cases in this study may not be sufficient to draw definitive conclusions. Additionally, the favorable outcomes are based on a single‐center study. To further validate these findings, larger case series, including multi‐center studies, are needed.

In conclusion, the TactiFlex catheter offers a promising combination of safety and efficacy for AF ablation, with high rates of first‐pass isolation and durable PVI lesions. Its innovative design facilitates consistent results regardless of operator experience, making it a valuable tool in the treatment of AF.

## CONFLICT OF INTEREST STATEMENT

Dr. Okumura belongs to the endowed departments of Boston Scientific Japan, Biotronik Japan, Abbott Medical Japan, Japan Lifeline, and Medtronic Japan.
